# Organoid‐Based Fibrosis Model of Endometrial Epithelium: Insights Into Intrauterine Adhesion Development

**DOI:** 10.1111/jcmm.70860

**Published:** 2025-09-28

**Authors:** Xinyu Qiao, Xin Huang, Yuchan Zhong, Ruiying Wang, Yujing Li, Fangyuan Li, Wenjie Bo, Jiagui Liang, Chang Liu, Wei Huang

**Affiliations:** ^1^ Department of Reproductive Medicine West China Second University Hospital of Sichuan University Chengdu China; ^2^ Key Laboratory of Birth Defects and Related Diseases of Women and Children of Ministry of Education Chengdu China; ^3^ NHC Key Laboratory of Chronobiology of Sichuan University Chengdu China; ^4^ Department of Reproductive Endocrinology West China Second University Hospital, Sichuan University Chengdu China

**Keywords:** endometrium, fibrosis, intrauterine adhesion, organoid, TGF‐β1

Dear Editor,

The human endometrium is a dynamic tissue lining the uterine cavity that undergoes hormonally regulated cycles of shedding, repair, and remodelling throughout the menstrual cycle and pregnancy [1]. Damage to the endometrial lining can lead to intrauterine adhesion (IUA), a condition where fibrous adhesions fuse the opposing uterine walls. This fusion may partially or completely obliterate the uterine cavity, causing menstrual abnormalities, reduced fertility, and recurrent pregnancy loss [2].

The pathology of IUA is characterised by endometrial fibrosis and scarring. This condition is underpinned by varying degrees of damage to the basal layer, glandular atrophy, and the development of an avascular, hypoxic stromal tissue [3]. Disentangling the mechanisms of endometrial fibrosis and developing effective regeneration strategies requires advanced experimental models. Among these, endometrial epithelial organoids (EEOs)—self‐organising, genetically stable 3D cultures—have emerged as a powerful system due to their long‐term expandability, cryopreservation potential, and hormone responsiveness [4, 5]. While these organoid models are valuable tools for investigating endometrial remodelling and receptivity, their application in the context of IUA has been limited. Specifically, research has focused on using organoids for regenerative therapies rather than as models to investigate the fibrotic pathogenesis of the disease itself [6, 7].

To address this gap, we pioneered a fibrotic EEOs model by inducing fibrosis with TGF‐β1. TGF‐β1 is a key driver of fibrosis in intestinal and lung organoid models, but its role in inducing a fibrotic phenotype in EEOs remained uncharacterised. Our work developed this platform as a novel in vitro model to investigate the pathology of IUA.

We established EEOs by isolating epithelial cells from fresh endometrial tissues of healthy donors undergoing diagnostic hysteroscopic procedures. Following mechanical dissociation and enzymatic digestion, we suspended the isolated cells in Matrigel and cultured them in a growth medium (Figure 1A). The methods and materials can be found in the Appendix S1 and Table S1‐S3. The cells began to self‐organise within 48 h, and by day 6, the cultures exhibited a 96% organoid formation rate, with individual organoids measuring 200–500 μm in diameter (Figure S1A). Successive passaging (to P3) progressively reduced stromal cells, immune cells, and erythrocyte content while enhancing 3D architectural maturation. The mature organoids formed distinct gland‐like structures with a central lumen. Furthermore, immunofluorescence staining confirmed their endometrial epithelial identity through robust expression of E‐cadherin (Figure S1B).

**FIGURE 1:**
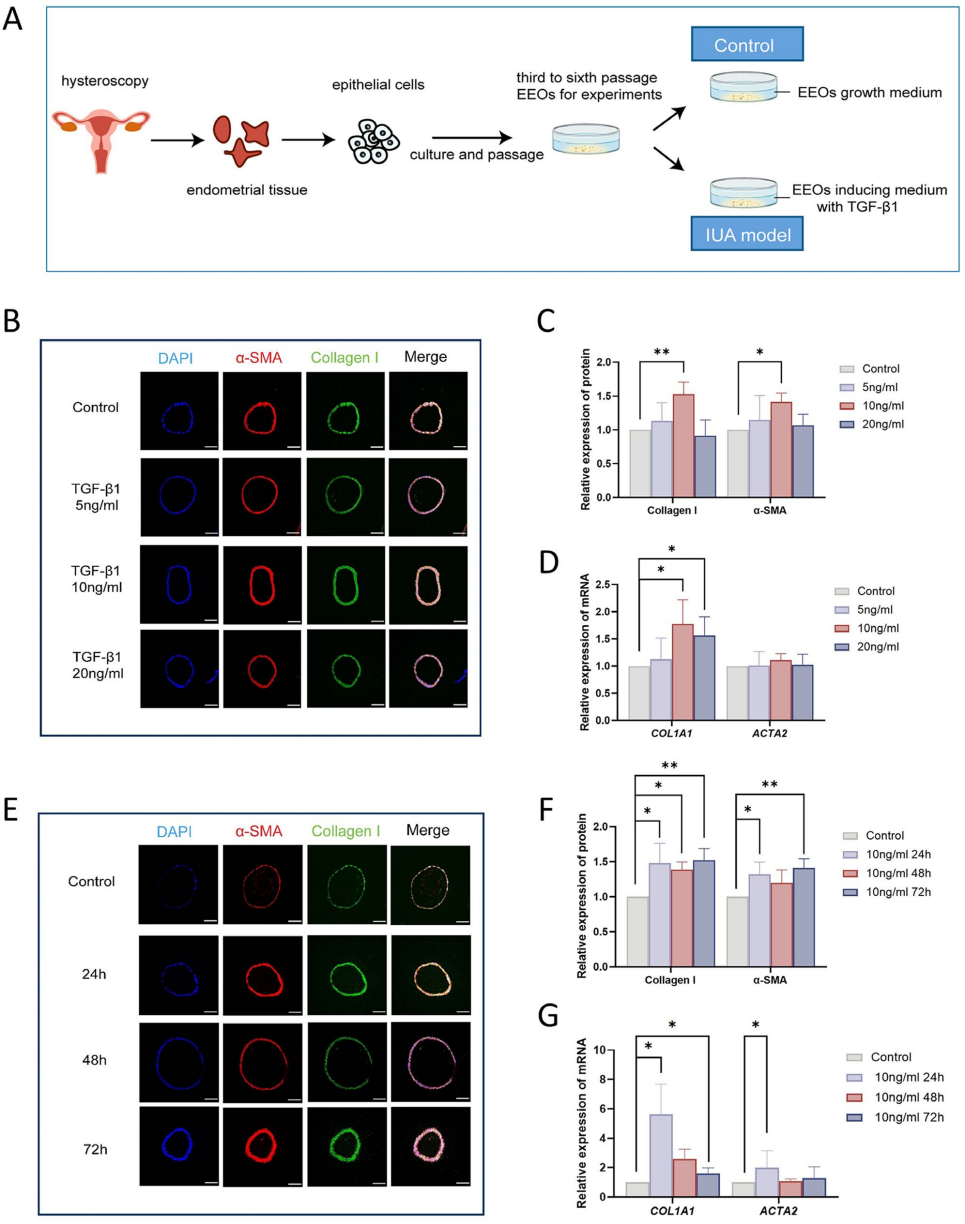
TGF‐β1 induces a fibrotic phenotype in endometrial epithelial organoids (EEOs). (A) Schematic overview of the protocol for generating EEOs from primary human endometrial tissue. (B) Representative immunofluorescence for α‐SMA (red) and Collagen I (green) in EEOs treated with different concentrations of TGF‐β1 (5, 10 and 20 ng/mL). Scale bars, 50 μm. (C) Quantification of α‐SMA and Collagen I fluorescence intensity. (D) RT‐qPCR analysis of *ACTA2* and *COL1A1* mRNA levels in EEOs treated with different concentrations of TGF‐β1. (E) Representative immunofluorescence for α‐SMA (red) and Collagen I (green) in EEOs treated with TGF‐β1 at a concentration of 10 ng/mL for 24, 48 and 72 h. Scale bars, 50 μm. (F) Quantification of α‐SMA and Collagen I fluorescence intensity. (G) RT‐qPCR analysis of *ACTA2* and *COL1A1* mRNA levels in EEOs treated with different durations of TGF‐β1. Data are presented as mean ± SD. Significance was determined by an independent‐sample *t*‐test. *p < 0.05, **p < 0.01, ***p < 0.001.

TGF‐β1 is a key driver of fibrosis in many organs, where it promotes extracellular matrix (ECM) accumulation and fibroblast‐to‐myofibroblast differentiation. Based on this role, we sought to establish a model of fibrosis relevant to IUA. To optimise the pro‐fibrotic conditions, we exposed EEOs to varying concentrations of TGF‐β1 (5, 10 or 20 ng/mL) for 72 h. This experiment revealed morphological defects and growth arrest within 24 h (Figure S1C). Based on immunofluorescence analysis for the fibrosis markers α‐SMA and Collagen I, we identified 10 ng/mL as the optimal concentration for inducing a robust fibrotic response (Figure 1B,C), a finding corroborated by RT‐qPCR (Figure 1D). A subsequent time‐course experiment using this optimal dose confirmed that significant fibrotic changes were established as early as 24 h post‐treatment (Figure 1E–G).

To define the molecular signature of this TGF‐β1‐induced phenotype, we performed comprehensive transcriptomic profiling of fibrotic EEOs (10 ng/mL TGF‐β1 for 24 h) versus controls. This analysis identified 1504 differentially expressed genes (DEGs), with 961 genes upregulated and 543 downregulated in the fibrotic organoids (Figure 2A,B; Figure S2A,B). Gene Ontology (GO) analysis of the upregulated genes revealed a significant enrichment in processes central to cell injury and ECM remodelling, such as response to wounding, ECM organisation, epithelial cell migration, positive regulation of cell adhesion, tissue remodelling, and collagen‐containing ECM (Figure 2C). KEGG pathway analysis further implicated that the upregulated DEGs in TGF‐β1‐treated EEOs were principally enriched in key pro‐fibrotic signalling cascades, such as ECM receptor interaction, PI3K‐AKT, Hippo, Wnt, and JAK–STAT signalling pathways (Figure 2D). Conversely, the downregulated transcripts were significantly related to the cell cycle, amino acid metabolism, DNA replication, and glycolysis/gluconeogenesis (Figure S2C,D). These findings, which were further supported by Gene Set Enrichment Analysis (GSEA) (Figure 2E), indicate a fundamental transcriptomic shift away from homeostasis and towards a pathological, fibrotic state.

**FIGURE 2:**
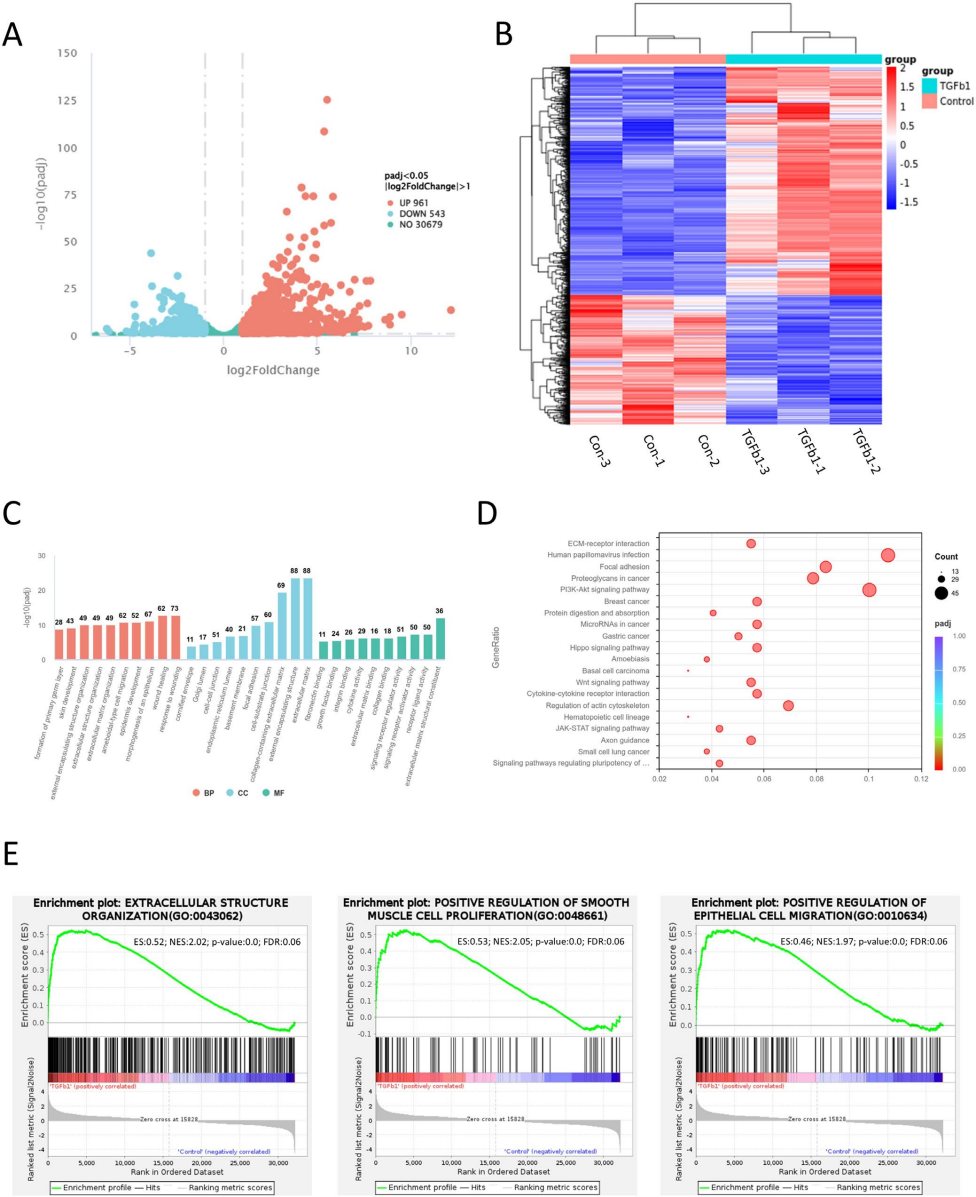
Transcriptomic profiling reveals a fibrotic signature in TGF‐β1‐treated endometrial epithelial organoids (EEOs). (A) Volcano plot of differentially expressed genes (DEGs) between TGF‐β1‐treated (10 ng/mL, 24 h) and control EEOs (|log_2_FC| > 1, *p*adj < 0.05). (B) Heatmap showing hierarchical clustering of the 1504 DEGs, demonstrating distinct expression profiles between TGF‐β1‐treated groups and control. (C) Gene Ontology (GO) enrichment analysis of upregulated DEGs, highlighting key terms associated with ECM organisation, wounding, and tissue remodelling. (D) KEGG pathway analysis of upregulated DEGs, identifying enrichment in pro‐fibrotic signalling pathways. (E) Gene Set Enrichment Analysis (GSEA) plots for representative gene sets significantly enriched in TGF‐β1‐treated EEOs: Extracellular structure organisation (GO: 0043062), positive regulation of smooth muscle cell proliferation (GO: 0048661), and positive regulation of epithelial cell migration (GO: 0010634).

Taken together, our results demonstrate that TGF‐β1 treatment (10 ng/mL for 24 h) effectively induces fibrosis in EEOs. The resulting transcriptomic profile—characterised by the activation of ECM remodelling and fibrotic signalling pathways—closely recapitulates the known pathological features of clinical IUA [8]. This work therefore establishes a valuable in vitro platform for investigating mechanistic studies and therapeutic screening in endometrial fibrosis.

## Author Contributions

**Xinyu Qiao:** writing – original draft, data curation, investigation, methodology. **Xin Huang:** data curation, writing – review and editing. **Yuchan Zhong:** methodology. **Ruiying Wang:** methodology. **Yujing Li:** methodology. **Fangyuan Li:** writing – review and editing. **Wenjie Bo:** data curation. **Jiagui Liang:** data curation. **Chang Liu:** data curation. **Wei Huang:** conceptualization, writing – review and editing, funding acquisition, supervision, project administration.

## Ethics Statement

This study received approval from the Ethics Committee of West China Second Hospital, Sichuan University (Approval No. 267–2025). All participants provided written informed consent prior to their inclusion in the study.

## Consent

All authors reviewed the final manuscript and consented to its publication.

## Conflicts of Interest

The authors declare no conflicts of interest.

## Supporting information


**Figure S1:** Generation, characterisation and induction of endometrial epithelial organoids (EEOs). (A) Representative brightfield micrograph of mature EEOs exhibiting complex, gland‐like structures in culture. Scale bar, 5 mm and 500 μm. (B) Immunofluorescence staining of a mature organoid confirms robust expression of the epithelial marker E‐cadherin (red). Nuclei are counterstained with DAPI (blue). Scale bar, 50 μm. (C) Representative brightfield images showing morphological changes in EEOs following treatment with TGF‐β1 (10 ng/mL, 72 h). Scale bar, 200 μm.

**Figure S2:** Quality control and analysis of downregulated genes from RNA‐Seq. (A) Venn diagram illustrating the number of expressed genes unique to and shared between control and TGF‐β1‐treated groups. (B) Principal Component Analysis (PCA) of transcriptomic data, demonstrating clear clustering and separation between control (black) and TGF‐β1‐treated (blue) samples. (C) GO enrichment analysis for downregulated DEGs, showing significant enrichment in terms related to the cell cycle and DNA replication. (D) KEGG pathway analysis for downregulated DEGs, highlighting pathways associated with the cell cycle and cellular metabolism.

**Table S1:** Components of the endometrial epithelial organoids (EEOs) wash medium.
**Table S2:** Components of the endometrial epithelial organoids (EEOs) growth and inducing medium.
**Table S3:** The primer sequences.

**Appendix S1:** Methods.

## Data Availability

The datasets and custom source code generated and analysed during the current study are not yet publicly deposited due to the ongoing nature of the research. However, they are available from the corresponding author on reasonable request.
