# Supplementary figures and images for: Organoid‐Based Fibrosis Model of Endometrial Epithelium: Insights Into Intrauterine Adhesion Development
Source: J Cell Mol Med. 2025 Sep 28;29(18):e70860. doi: 10.1111/jcmm.70860 (PMC12476959; doi:10.1111/jcmm.70860)

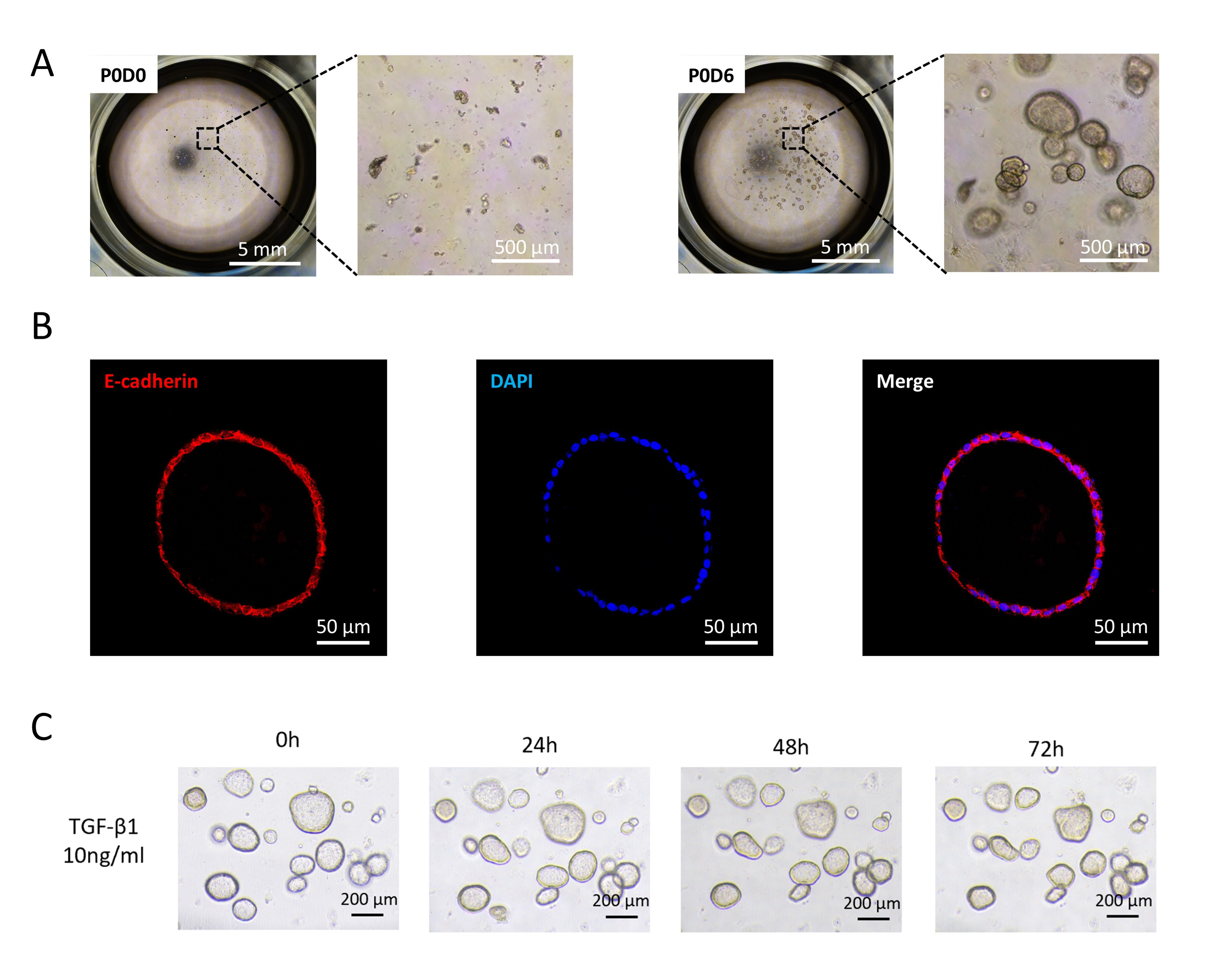

Supplement: Supplementary file 1 — Figure S1: Generation, characterisation and induction of endometrial epithelial organoids (EEOs). (A) Representative brightfield micrograph of mature EEOs exhibiting complex, gland‐like structures in culture. Scale bar, 5 mm and 500 μm. (B) Immunofluorescence staining of a mature organoid confirms robust expression of the epithelial marker E‐cadherin (red). Nuclei are counterstained with DAPI (blue). Scale bar, 50 μm. (C) Representative brightfield images showing morphological changes in EEOs following treatment with TGF‐β1 (10 ng/mL, 72 h). Scale bar, 200 μm. [file JCMM-29-e70860-s001.tif]

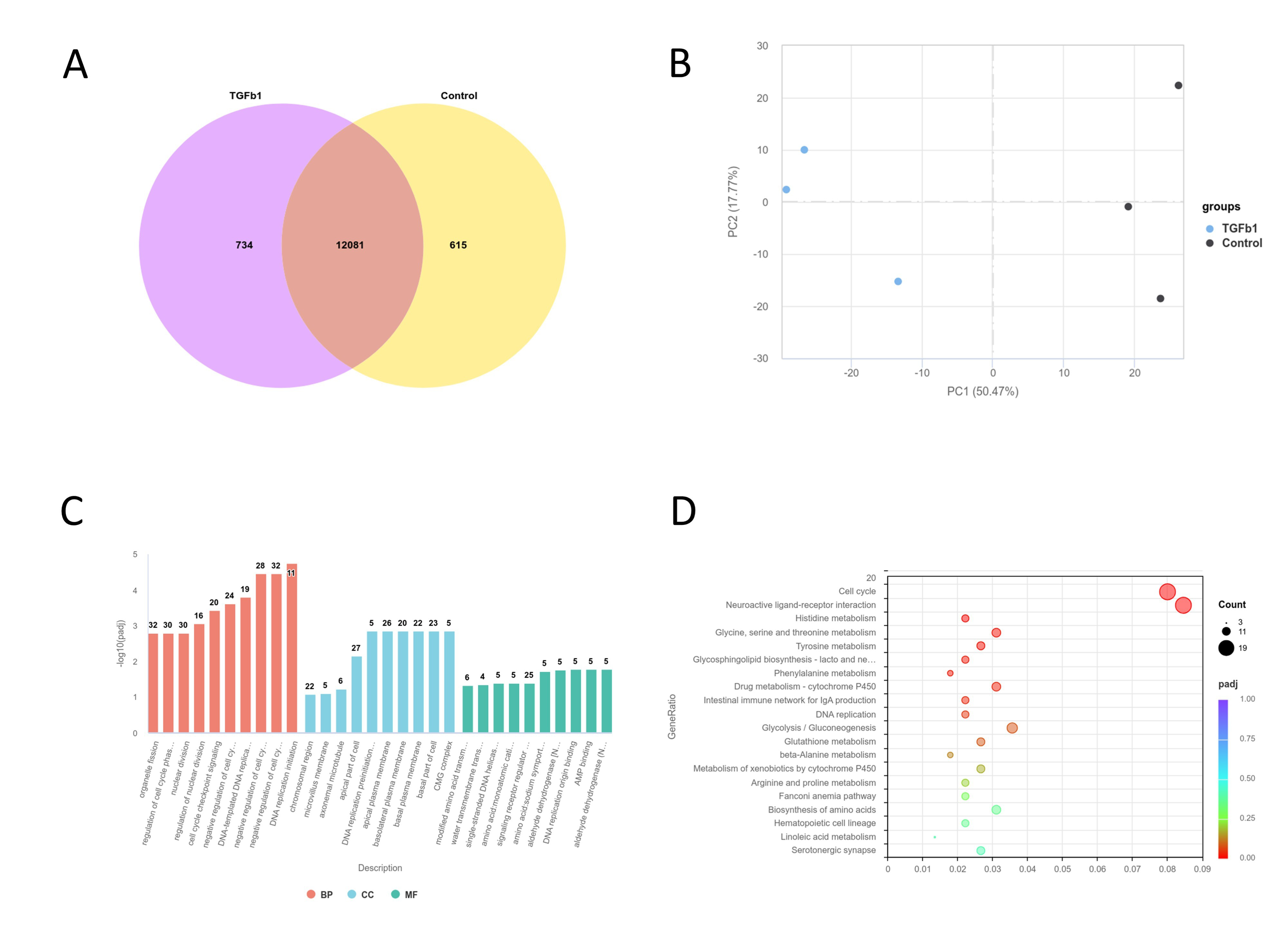

Supplement: Supplementary file 2 — Figure S2: Quality control and analysis of downregulated genes from RNA‐Seq. (A) Venn diagram illustrating the number of expressed genes unique to and shared between control and TGF‐β1‐treated groups. (B) Principal Component Analysis (PCA) of transcriptomic data, demonstrating clear clustering and separation between control (black) and TGF‐β1‐treated (blue) samples. (C) GO enrichment analysis for downregulated DEGs, showing significant enrichment in terms related to the cell cycle and DNA replication. (D) KEGG pathway analysis for downregulated DEGs, highlighting pathways associated with the cell cycle and cellular metabolism. [file JCMM-29-e70860-s002.tif]
