# Supplementary material for: Organoid‐Based Fibrosis Model of Endometrial Epithelium: Insights Into Intrauterine Adhesion Development
Source: J Cell Mol Med. 2025 Sep 28;29(18):e70860. doi: 10.1111/jcmm.70860 (PMC12476959; doi:10.1111/jcmm.70860)
Supplement: Supplementary file 3 — Table S1: Components of the endometrial epithelial organoids (EEOs) wash medium. Table S2: Components of the endometrial epithelial organoids (EEOs) growth and inducing medium. Table S3: The primer sequences. [file JCMM-29-e70860-s004.docx]

Table S1 Components of the Endometrial epithelial organoids (EEOs) wash medium.

| Component of Wash medium | Dilution | Source |
| --- | --- | --- |
| Basal medium | Advanced DMEM/F12 | 12634010, Gibco |
| Penicilin/Streptomycin | 1% | 15140122, Gibco |
| Glutamax | 1% | 35050061, Gibco |
| Hepes | 1% | 15630080, Gibco |

Table S2 Components of the Endometrial epithelial organoids (EEOs) growth and inducing medium.

| Component of expansion medium | Dilution | Source |
| --- | --- | --- |
| Basal medium | Advanced DMEM/F12 | 12634010, Gibco |
| Penicilin/Streptomycin | 1% | 15140122, Gibco |
| Glutamax | 1% | 35050061, Gibco |
| Hepes | 1% | 15630080, Gibco |
| EGF | 50 ng/ml | 236-EG-200, R&D |
| FGF10 | 20 ng/ml | 100-26-1mg, PeproTech |
| Noggin | 100 ng/ml | 6057-NG-100, R&D |
| R-spondin1 | 250ng/ml | 861-RS1-1000, biogenous |
| A83-01 | 500nM | s7692, Selleck |
| SB202190 | 500nM | 8158S, CST |
| Y27632 | 10 µM | s1049, Selleck |
| B27 | 1% | 17504044, Gibco |
| Nicotinamide | 2 mM | N0636, sigma |
| N-acetyl L-cysteine | 1.25 mM | A9165, sigma |

Table S3 The primer sequences.

| Primer name  (Human) | 5′-3′ |
| --- | --- |
| GAPDH | F: TGCACCACCAACTGCTTAGC  R: GGCATGGACTGTGGTCATGAG |
| Col1a1 | F: TTGTGCGATGACGTGATCTGT  R: TTGGTCGGTGGGTGACTCTG |
| Acta2 | F: CGTTACTACTGCTGAGCGTG  R: TGAAGGATGGCTGGAACAGG |
