# Supplementary material for: Organoid‐Based Fibrosis Model of Endometrial Epithelium: Insights Into Intrauterine Adhesion Development
Source: J Cell Mol Med. 2025 Sep 28;29(18):e70860. doi: 10.1111/jcmm.70860 (PMC12476959; doi:10.1111/jcmm.70860)
Supplement: Supplementary file 4 — Appendix S1: Methods. [file JCMM-29-e70860-s003.docx]

**Methods**

**Patient samples**

This study was approved by the Ethics Committee of West China Second University Hospital (Approval No. 267-2025). All patients enrolled signed an informed consent before being enrolled in the study. The patient consents to the collection of tissue samples and clinical information without prejudice to the pathological diagnosis. Endometrial tissue was obtained during outpatient hysteroscopy.

**Establishment and maintenance of Endometrial epithelial organoids (EEOs)**

Fresh endometrial tissue samples from healthy donors were subsequently immersed in a dulbecco's modified eagle medium/F-12 (DMEM/F-12) (Gibco, 11330032) supplemented with 4% penicillin and streptomycin (Gibco, 15140122). Tissue is minced into fragments (1–2 mm³) and washed to remove blood and debris under sterile conditions. Then, the fragments are mechanically and enzymatically digested using DMEM/F-12 contained 2 mg/mL collagenase (sigma, C4-28-100MG), 0.01% trypsin (Gibco, 25200072) , and 40 μg/mL Type I DNase (sigma, D5025-150KU) at 37°C for 20-30 minutes with gentle agitation every 10 min. The digestion process was halted by adding an equal volume of 10% fetal bovine serum (FBS)/DMEM/F-12 mixture. The digested solution was subsequently passed through 100 and 40 μm cell sieves in sequence. The 40 μm cell sieve was backwashed using DMEM/F-12, and the resulting solution was collected and subjected to centrifugation at 300g for 5 min, then resuspend precipitate with 1ml of wash medium (Supporting Information, Table S1) and centrifuge at 200g for 3 minutes, repeat three times with wash medium, and discard the supernatant. Cell mass precipitation was embedded in Matrigel (Corning, 356231) to mimic the extracellular microenvironment and seeded into a 24-well suspension culture plate (30 μl/well). After solidification of the Matrigel, the expansion medium (500μl/well, Supporting Information, Table S2) was added to culture the organoids at 37°C with 5% CO₂. Medium was refreshed every 3-5 days. Passaging occurred every 8-11 days when the EOs diameter reached 200-500μm via mechanical and enzymatic digestion followed by re-embedding in fresh Matrigel.

**TGF-β1 inducing culture of EOs**

To mimic fibrosis conditions, the organoids were cultured in inducing medium (the expansion medium without A83 and Noggin) with different concentration of TGF-β1(PeproTech, 100-21-2ug) during 24, 48, and 72h.

**Immunofluorescence**

Prior to paraffin embedding, the EOs were removed from Matrigel using Wash Medium, fixed in 4% paraformaldehyde overnight and embedded into 3% agarose (Melford, MB1200). After paraffin embedding, paraffin sections of 4 μm were cut with a microtome and collected on adhesive slides. Immunofluorescence began with dewaxing (xylene, graded ethanol), antigen retrieval (EDTA buffer, 95°C, 30 min), and blocking (5% BSA, 20 min). Primary antibodies (diluted in 5% BSA) were incubated overnight at 4 °C. Primary antibodies used included α-SMA (1：1000, ab7817, Abcam), Collagen I (1:500, 14695-1-AP, Proteintech), Vimentin (1:200, 10366-1-AP, Proteintech), E-Cadherin (1:1000, ab231303, Abcam). Then, the section was washed three times with PBS, and incubated with secondary antibodies 488/594 (1:500) at room temperature for 1 h, followed by staining with DAPI (1:2000, Solarbio, China) to label the nucleus. The stained cells were observed using a laser scanning confocal microscope (Olympus, FV3000). The fluorescence images were analyzed using ImageJ Plus software.

**Real-time quantitative PCR analysis**

Total RNA was extracted from EOs using a RNAprep Pure Micro Kit (DP420, TIANGEN). Total RNA (1 μg) from each sample was reverse transcribed using PrimeScript™ RT reagent Kit (RR047A, TaKaRa). Real-time quantitative PCR (RT-qPCR) was performed using Hieff qPCR SYBR Green Master Mix (11201ES50, YEASEN) on the Lightcycler 96 real-time PCR system (Roche). The primer sequences are listed in Supporting Information, Table S3. Gene expression levels were normalized to an internal control, and relative expression was calculated using the 2−ΔΔCt method.

**RNA-seq transcriptome analysis**

The quality and concentration of RNA extracted from EOs were determined using a Agilent 2100 bioanalyzer. For sequencing library construction, enrichment of mRNA from total RNA was carried out using oligo (dT) magnetic beads. The sequencing library is then quality-controlled using Qubit, Bioanalyzer, and qPCR. The qualified libraries underwent Next Generation Sequencing(NGS) utilizing Illumina's Sequencing Technology by Synthesis, wherein fluorescence detection was employed for nucleotide identification during the synthesis of the complementary chain. Subsequent bioinformatics analysis includes raw data processing with fastp to obtain clean reads, reference genome alignment using HISAT2 (v2.2.1), gene expression quantification via featureCounts (v2.0.6) and FPKM normalization, and differential expression analysis with DESeq2 R package (v1.42.0). ClusterProfiler R package (v4.8.1) was used to perform Gene Ontology (GO) and Kyoto Encyclopedia of Genes and Genomes (KEGG) Pathway analysis on differentially expressed genes (DEGs) , applying log2 fold change >1 and adjusted P-value 0.05. The Gene Set Enrichment Analysis (GSEA) was performed using a local version of the analysis tool (http://www.broadinstitute.org/gsea/index.jsp), with independent analytical runs conducted for the GO and KEGG datasets.

**Statistical analysis**

Data were reported as mean and standard deviation (SD) for continuous variables with normal distribution (assessed by Shapiro-Wilk test). Statistical differences between two groups were assessed by independent samples t-test. All data were analyzed using SPSS Statistics 26.0 software (IBM) and differences were assumed significant when p < 0.05 (∗ and ** represent p < 0.05 and p < 0.01, respectively).
